# A pitfall of using the circular‐edge technique with image averaging for spatial resolution measurement in iteratively reconstructed CT images

**DOI:** 10.1002/acm2.12821

**Published:** 2020-01-20

**Authors:** Akihiro Narita, Masaki Ohkubo

**Affiliations:** ^1^ Graduate School of Health Sciences Niigata University Chuo‐ku Niigata Japan

**Keywords:** circular‐edge technique, computed tomography, iterative reconstruction algorithm, modulation transfer function, spatial resolution

## Abstract

The circular‐edge technique using a low‐contrast cylindrical object is commonly used to measure the modulation transfer functions (MTFs) in computed tomography (CT) images reconstructed with iterative reconstruction (IR) algorithms. This method generally entails averaging multiple images of the cylinder to reduce the image noise. We suspected that the cylinder edge shape depicted in the IR images might exhibit slight deformation with respect to the true shape because of the intrinsic nonlinearity of IR algorithms. Image averaging can reduce the image noise, but does not effectively improve the deformation of the edge shape; thereby causing errors in the MTF measurements. We address this issue and propose a method to correct the MTF. We scanned a phantom including cylindrical objects with a CT scanner (Ingenuity Elite, Philips Healthcare). We obtained cylinder images with iterative model reconstruction (IMR) algorithms. The images suggested that the depicted edge shape deforms and fluctuates depending on slice positions. Because of this deformation, image averaging can potentially cause additional blurring. We define the deformation function *D* that describes the additional blurring, and obtain *D* by analyzing multiple images. The MTF measured by the circular‐edge method (referred to as MTF') can be thought of as the multiplication of the true MTF by the Fourier transformation (FT) of *D*. We thus obtain the corrected MTF (MTF*_corrected_*) by dividing MTF' by the FT of *D*. We validate our correction method by comparing the calculated images based on the convolution theorem using MTF' and MTF*_corrected_* with the actual images obtained with the scanner. The calculated image using MTF*_corrected_* is more similar to the actual image compared with the image calculated using MTF', particularly in edge regions. We describe a pitfall in MTF measurement using the circular‐edge technique with image averaging, and suggest a method to correct it.

## 1 INTRODUCTION

Iterative reconstruction (IR) algorithms have been widely implemented for clinical computed tomography (CT). IR methods can reduce image noise, which is mainly caused by radiation quantum fluctuation in the CT projection data, while maintaining (or improving) the spatial resolution.^1^, ^2^, ^3^, ^4^ Most IR algorithms incorporate statistical models (photon and noise statistics) and the scanner geometry and optics, and have nonlinear properties.^5^, ^6^ It has been reported that the nonlinear properties cause spatial resolution variability depending on image noise levels and object contrast.^7^, ^8^, ^9^ Therefore, the modulation transfer function (MTF), one of the most comprehensive metrics for spatial resolution, measured using traditional approaches with high contrast wires or beads, is not applicable for characterizing the spatial resolution of clinical IR images. Richard et al. developed a new MTF measurement approach, called the “circular‐edge technique,” using a low‐contrast cylindrical object.^7^ This technique has been widely used for MTF measurements of IR images. Most of the studies using this technique computed the average of the consecutive cross‐sectional images of the cylinder and/or the average of many images acquired from repeated scans to improve the signal‐to‐noise ratio.^1^, ^9^, ^10^, ^11^, ^12^

Because of the intrinsic nonlinearity of IR algorithms, the resulting image properties are complicated compared with those of filtered back projection (FBP) images. Leipsic et al.^13^ reported that in cardiac CT angiography, reconstructions obtained using adaptive statistical iterative reconstruction (ASIR) differ in appearance from traditional FBP images, exhibiting a different noise texture and smoothed borders. Singh et al.^14^ observed a step‐like artifact at tissue interfaces (such as the margins of the liver, spleen, and blood vessels) in abdominal CT images reconstructed using ASIR. The imaging at border/edge regions using IR algorithms is potentially sensitive to slight fluctuations in the CT projection data, including noise. In a phantom study, Li et al.^9^ obtained multiple IR images using repeated scans, and assessed the standard deviation of CT values locally in the edge regions of circular objects. They considered this standard deviation as “edge‐noise,” and found that the edge‐noise was greater than the standard deviation computed for uniform regions; thereby suggesting a specific anomaly in edge regions. The object edge shape depicted in IR images may deform slightly with respect to the ideal shape (circle) and fluctuate in repeated scans; this effect is one potential reason for the increased edge‐noise. Averaging multiple images can reduce the image noise, but does not effectively improve the deformation and fluctuation of the object edge shape depicted in the IR images. When applying image averaging with the circular‐edge technique, the occurrence of edge shape deformation may adversely affect MTF measurements.

The aim of this study is to address this issue and propose a method to correct the MTF measured using the circular‐edge technique. To verify the validity of the proposed method, we compared the computed images obtained by applying the convolution theorem using the corrected MTF with the true images obtained by the CT scan.

## 2 MATERIALS AND METHODS

### 2.A Equipment and imaging parameters

We used the sensitometry module (CTP404) included with the Catphan 600 phantom (The Phantom Laboratory, Salem, NY). The module consists of eight cylindrical objects; we used two objects made from Delrin (approximately 350 HU at 120 kVp) and polystyrene (PS) (approximately −30 HU at 120 kVp). The background CT value was approximately 100 HU at 120 kVp. We placed the phantom in the center of the scanner field of view (FOV) such that the cylinder was parallel to the *z* direction, and therefore perpendicular to the *x*–*y* scanning plane. We scanned the phantom with a multidetector row CT scanner (Ingenuity Elite, Philips Healthcare, Netherland) at 120 kVp, 100 mA, with a one‐second/rotation, a pitch of 1.17, and detector configuration of 16 × 0.625 mm. Twenty consecutive cross‐sectional images along the cylinder were reconstructed at a 200 mm FOV, with a 1‐mm slice thickness and a 1‐mm interval. The scan and reconstruction was repeated 10 times, resulting in a total of 200 images. The image reconstruction was performed using the FBP algorithm and the iterative model reconstruction (IMR) algorithms *Body Routine* and *Body SharpPlus*. IMR algorithms feature three noise reduction levels (level 1–3), where level 3 provides the maximum noise reduction. We used levels 1 and 3 for both IMR algorithms, and obtained 200 images with each.

### 2.B Pitfall of circular‐edge technique used with image averaging

**Figure 1:**
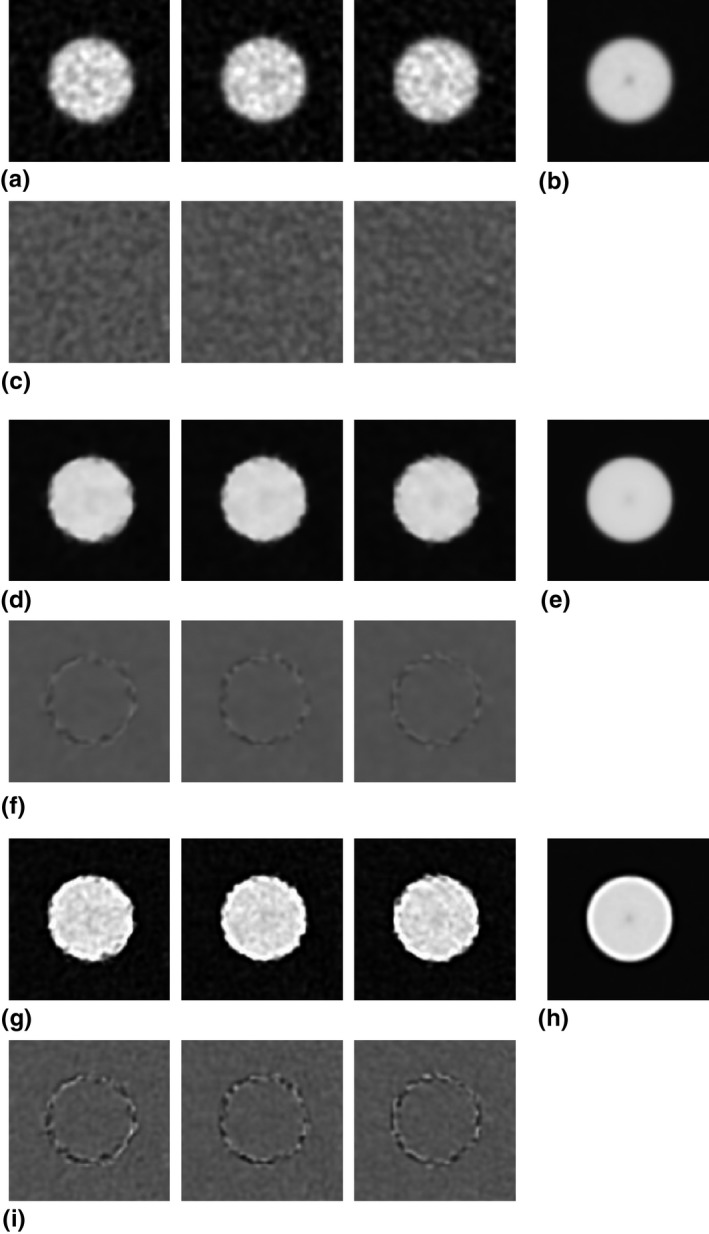
(a) Three example computed tomography (CT) cross‐sectional images of a cylindrical object reconstructed using the filtered back projection (FBP) algorithm. (b) The mean image obtained by averaging all FBP images. (c) The images obtained by subtracting the mean image (b) from each slice image (a). (d–f) Results obtained using IMR *Body Routine* level 3. Images are analogous to those in (a–c). (g–i) Results obtained using iterative model reconstruction *SharpPlus* level 3. The images are analogous to those in (a–c). The window settings are WL/WW = −225/310 HU for images in (a, b, d, e, g, h), and 0/200 HU for images in (c, f, i).

Figure 1(a) shows three adjacent cross‐sectional images of the phantom obtained using FBP. We obtained a mean image by averaging overall 200 images reconstructed using FBP [Fig. 1(b)], and subtracted the mean image from each slice image [Fig. 1(c)]. The adjacent FBP images were slightly different depending on the slice positions; we attribute these differences to the image noise because the subtraction images showed uniform noise overall, without indicating cylinder edge residuals. In the same way, the corresponding images when using IMR *Body Routine* level 3 and *Body SharpPlus* level 3 are shown in Figs. 1(d)–1(f) and 1(g)–1(i), respectively. In the adjacent IMR (*Body Routine* and *Body SharpPlus*) images, the subtraction images [Figs. 1(f) and 1(i)] show not only the overall noise, but also the cylinder edge residuals. This is particularly evident in the *Body SharpPlus* images. Some deformation from the ideal object shape (circle) might be implicit in the IMR images, depending on slice positions. This deformation may cause an error in the measurement of spatial resolution by the circular‐edge technique using the average of multiple images. We address this issue as follows.

A CT image is characterized by the spatial resolution of the system. When considering a CT image of a uniform cylindrical object placed parallel to the *z* direction (perpendicular to the *x*–*y* scanning plane), the resulting image is expressed as follows:^15^, ^16^, ^17^

(1) $$I\left(x,y\right)=O\left(x,y\right)*\text{PSF}\left(x,y\right),$$

where $O\left(x,y\right)$ is an object function of a circular shape with uniform density, and $\text{PSF}\left(x,y\right)$ is the two‐dimensional (2D) point spread function (PSF). The operator * is the 2D convolution. Because of the uniform circular shape of the cylinder, the cross‐sectional image $I\left(x,y\right)$ does not change with the slice position along the *z*‐axis. However, we observed differences between consecutive slice images reconstructed using IMR (Fig. 1). To describe a practical image generation system that includes the object‐shape deformation present in IMR images, we make several assumptions and modify Eq. (1) as follows.

First, we include a deformation between each of the cross‐sectional images and the original circular shape in the object function. Thus, we write Eq. (1) as follows:

(2) $$I_{i}^{{}^{\prime }}\left(x,y\right)=O_{i}^{{}^{\prime }}\left(x,y\right)*\text{PSF}\left(x,y\right),$$

where $I_{i}^{{}^{\prime }}\left(x,y\right)$={$I_{1}^{{}^{\prime }}$, $I_{2}^{{}^{\prime }}$,..., $I_{n}^{{}^{\prime }}$} are consecutive images at different positions along the *z*‐axis (*i* is the slice number and *n* is the total number of slices), and $O_{i}^{{}^{\prime }}\left(x,y\right)=\left\{O_{1}^{{}^{\prime }},O_{2}^{{}^{\prime }},\ldots ,O_{n}^{{}^{\prime }}\right\}$ are the object functions including deformations from an ideal circle; we refer to $O_{i}^{{}^{\prime }}\left(x,y\right)$ as effective object functions. Each $I_{i}^{{}^{\prime }}\left(x,y\right)$ is formed from a corresponding $O_{i}^{{}^{\prime }}\left(x,y\right)$, thereby accounting for the IMR image differences present in Fig. 1. The circular‐edge technique used for the MTF measurement typically includes using the average of multiple cross‐sectional images of the cylinder to reduce the image noise. This averaging corresponds to the averaging of $I_{i}^{{}^{\prime }}\left(x,y\right)$, and Eq. (2) is expressed as follows:

(3) $$\bar{I_{i}^{{}^{\prime }}}\left(x,y\right)=\bar{O_{i}^{{}^{\prime }}}\left(x,y\right)*\text{PSF}\left(x,y\right),$$

where $\bar{I_{i}^{{}^{\prime }}}\left(x,y\right)$ is a mean image obtained by averaging overall $I_{i}^{{}^{\prime }}\left(x,y\right)$, and $\bar{O_{i}^{{}^{\prime }}}\left(x,y\right)$ is the mean effective object function obtained by averaging overall $O_{i}^{{}^{\prime }}\left(x,y\right)$.

Next, we assume that the shape of $\bar{O_{i}^{{}^{\prime }}}\left(x,y\right)$ corresponds to a blurred circular shape with respect to an ideal circle (i.e., $O\left(x,y\right)$), because $\bar{O_{i}^{{}^{\prime }}}\left(x,y\right)$ is obtained by averaging the various deformations included in multiple effective object functions. Based on this assumption, we obtain.

(4) $$\bar{O_{i}^{{}^{\prime }}}\left(x,y\right)=O\left(x,y\right)*D\left(x,y\right),$$

where $D\left(x,y\right)$ is a blurring function whereby the blurring is originated from the deformations in $O_{i}^{{}^{\prime }}\left(x,y\right)$. Therefore, we refer to $D\left(x,y\right)$ as the deformation function. By applying Eqs. (4) to (3), we have

(5) $$\bar{I_{i}^{{}^{\prime }}}\left(x,y\right)=O\left(x,y\right)*D\left(x,y\right)*\text{PSF}\left(x,y\right).$$

The circular‐edge technique is based on Eq. (1) assuming the ideal circular shape of $O\left(x,y\right)$ and the isotropy of the in‐plane resolution; this provides an MTF that is equivalent to the Fourier transformation of $\text{PSF}\left(x,y\right)$. That is, the resultant MTF is written as follows:

(6) $$\text{MTF}\left(w\right)=\left|F\left[\text{PSF}\left(x,y\right)\right]\right|,$$

(7) $$w=\sqrt{u^{2}+v^{2}},$$

where *F* is the Fourier transform, *u* and *v* are the spatial frequency coordinates in the *x* and *y* directions, respectively, and *w* is spatial frequency in the radial direction. When applying image averaging with the circular‐edge technique, we assume Eq. (5) in place of Eq. (1). The $\text{PSF}\left(x,y\right)$ in Eq. (1) corresponds to the term of $D\left(x,y\right)*\text{PSF}\left(x,y\right)$ in Eq. (5); therefore, the circular‐edge technique would provide the following MTF, which we refer to as ${\text{MTF}}^{{}^{\prime }}\left(w\right)$:

(8) $${\text{MTF}}^{{}^{\prime }}\left(w\right)=\left|F\left[D\left(x,y\right)*\text{PSF}\left(x,y\right)\right]\right|=\left|F\left[D\left(x,y\right)\right]\right|\times \text{MTF}\left(w\right).$$

Thus, the circular‐edge method using image averaging provides not the true value $\text{MTF}\left(w\right)$, but instead the one multiplied by the deformation function $D\left(x,y\right)$; this leads to erroneous MTF measurements.

### 2.C MTF measurement using the circular‐edge technique with a correction

#### 2.C.1 MTF obtained using the circular‐edge technique with image averaging

Using a total of 200 slice images of the cylinder reconstructed with IMR *Body Routine* and *Body SharpPlus* (levels 1 and 3), we measured the MTFs using the circular‐edge technique according to conventional metrics^7^. First, we averaged over the 200 images to obtain a mean image. Next, we measured the pixel values (CT numbers) around the circular edge in the mean image and plotted them as a function of their distance from the center of the cylinder, thereby forming an ensemble edge spread function (ESF). Then, to create equidistant ESF data, we resampled the ensemble ESF with a bin width that was determined empirically by considering the tradeoff between noise reduction and resolution loss. After differentiating the resampled ESF to obtain the line spread function (LSF), we multiplied the LSF by a Hann window to remove the noise on both sides of the LSF (far from its center). From the resultant LSF, we obtained the MTF by the fast Fourier transform; we consider this MTF as the ${\text{MTF}}^{{}^{\prime }}\left(w\right)$ in Eq. (8).

#### 2.C.2 MTF correction method

**Figure 2:**
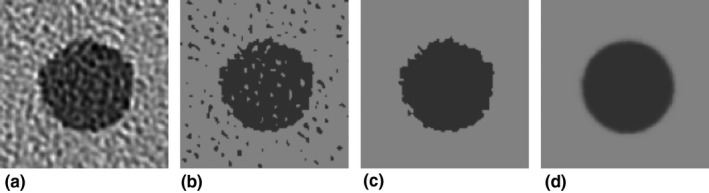
(a) An example of a computed tomography image of cylindrical polystyrene (PS) object reconstructed using the IMR *Body SharpPlus* (level 1) algorithm. (b) Binary image obtained from the image in (a) by thresholding. (c) Effective object function obtained from the image in (b). (d) The mean image obtained by averaging all of the 200 effective object functions. The window settings are WL/WW = 25/450 HU.

We measured the CT values of the cylindrical object and its surroundings in the phantom in the original images, and defined the results as CT_1_ and CT_2_, respectively. From the 200 images $I_{i}^{{}^{\prime }}\left(x,y\right)$ of the cylinder, we calculated the 200 corresponding effective object functions $O_{i}^{{}^{\prime }}\left(x,y\right)$. As an example, $I_{i=k}^{{}^{\prime }}\left(x,y\right)$ and $O_{i=k}^{{}^{\prime }}\left(x,y\right)$ (*k* is a slice number) are shown in Fig. 2. From $I_{i=k}^{{}^{\prime }}\left(x,y\right)$ [Fig. 2(a)], a binary image is obtained by simple thresholding with the mean value of CT_1_ and CT_2_ [Fig. 2(b)]. This threshold value corresponded to the half‐maximum of the full‐width‐at‐half‐maximum that is generally used to assess of the size/diameter of an object.^18^ $O_{i=k}^{{}^{\prime }}\left(x,y\right)$ is obtained by removing holes/noise in the foreground/background [Fig. 2(c)]; we used a simple flood‐fill operation with four‐connected neighborhoods and extracted the largest connected pixel region in the image. Then, we obtain $\bar{O_{i}^{{}^{\prime }}}\left(x,y\right)$ by averaging all 200 $O_{i}^{{}^{\prime }}\left(x,y\right)$ [Fig. 2(d)].

We applied the circular‐edge technique to obtain the frequency characteristics of the deformation function $D\left(x,y\right)$, which is the Fourier transformation of $D\left(x,y\right)$ in Eq. (4). The circular‐edge technique provides the MTF that corresponds to the Fourier transformation of $\text{PSF}\left(x,y\right)$ in Eq. (1). Comparing Eq. (4) with Eq. (1), the $\text{PSF}\left(x,y\right)$ in Eq. (1) corresponds to $D\left(x,y\right)$ in Eq. (4); therefore, using the circular‐edge technique, we can obtain the Fourier transformation of $D\left(x,y\right)$ using $\bar{O_{i}^{{}^{\prime }}}\left(x,y\right)$. The ${\text{MTF}}^{{}^{\prime }}\left(w\right)$ in Eq. (8) can be corrected using the Fourier transformation of $D\left(x,y\right)$. We refer to the corrected MTF as ${\text{MTF}}_{\mathrm{corrected}}\left(w\right)$, where:

(9) $${\text{MTF}}_{\mathrm{corrected}}\left(w\right)={\text{MTF}}^{{}^{\prime }}\left(w\right)/\left|F\left[D\left(x,y\right)\right]\right|.$$

With this correction, we can eliminate the effect of $D\left(x,y\right)$ on ${\text{MTF}}^{{}^{\prime }}\left(w\right)$, achieving an accurate MTF measurement.

### 2.D Validation of our MTF correction method

#### 2.D.1 **Validation using the averaged image** $\bar{I_{i}^{{}^{\prime }}}\left(x,y\right)$

To investigate the accuracy of the ${\text{MTF}}^{{}^{\prime }}\left(w\right)$ measured from an averaged image using the circular‐edge technique, we performed image simulations based on Eq. (3). Assuming the isotropy of the in‐plane resolution, we transform Eq. (3) as follows:^19^

(10) $$\bar{I_{i}^{{}^{\prime }}}\left(x,y\right)=F^{-1}\left\{F\left[\bar{O_{i}^{{}^{\prime }}}\left(x,y\right)\right]\cdot \text{MTF}\left(w\right)\right\}.$$

We substituted the measured ${\text{MTF}}^{{}^{\prime }}\left(w\right)$ for $\text{MTF}\left(w\right)$ in Eq. (10), used $\bar{O_{i}^{{}^{\prime }}}\left(x,y\right)$ as described in Section 2.C.2, and then calculated the image $\bar{I_{i}^{{}^{\prime }}}\left(x,y\right)$. When the measured ${\text{MTF}}^{{}^{\prime }}\left(w\right)$ was accurate, the calculated image suitably matched the actual IMR image obtained with the scanner. The difference between the calculated and actual images depended on the accuracy of the measured ${\text{MTF}}^{{}^{\prime }}\left(w\right)$; we quantified the difference in the images by the root mean‐squared error (RMSE). Using a 2‐mm‐thick ring‐shaped region of interest (ROI) that contained the edge of the cylinder, we obtained the RMSE between the calculated and the actual images in the ROI. In the same way, we used ${\text{MTF}}_{\mathrm{corrected}}\left(w\right)$ in place of ${\text{MTF}}^{{}^{\prime }}\left(w\right)$ in Eq. (10) to investigate the accuracy of ${\text{MTF}}_{\mathrm{corrected}}\left(w\right)$.

#### 2.D.2 **Validation using each image** $I_{i}^{{}^{\prime }}\left(x,y\right)$

Similarly to the approach described in Section 2.D.1, we investigated the accuracy of ${\text{MTF}}^{{}^{\prime }}\left(w\right)$ and ${\text{MTF}}_{\mathrm{corrected}}\left(w\right)$ using each image $I_{i}^{{}^{\prime }}\left(x,y\right)$ and the corresponding $O_{i}^{{}^{\prime }}\left(x,y\right)$. We transformed Eq. (2) as follows:^19^

(11) $$I_{i}^{{}^{\prime }}\left(x,y\right)=F^{-1}\left\{F\left[O_{i}^{{}^{\prime }}\left(x,y\right)\right]\cdot \text{MTF}\left(w\right)\right\}.$$

We substituted the measured ${\text{MTF}}^{{}^{\prime }}\left(w\right)$ for $\text{MTF}\left(w\right)$ in Eq. (11), and calculated a total of 200 slice images $I_{i}^{{}^{\prime }}\left(x,y\right)$ using the corresponding $O_{i}^{{}^{\prime }}\left(x,y\right)$ described in Section 2.C.2. We quantified the differences between the calculated and actual images by the RMSEs using a ring‐shaped ROI that includes the edge of the cylinder. In the same way, we used ${\text{MTF}}_{\mathrm{corrected}}\left(w\right)$ in place of ${\text{MTF}}^{{}^{\prime }}\left(w\right)$ in Eq. (11) to investigate the accuracy of ${\text{MTF}}_{\mathrm{corrected}}\left(w\right)$.

## 3 RESULTS

### 3.A MTF measurement using the circular‐edge technique with correction

**Figure 3:**
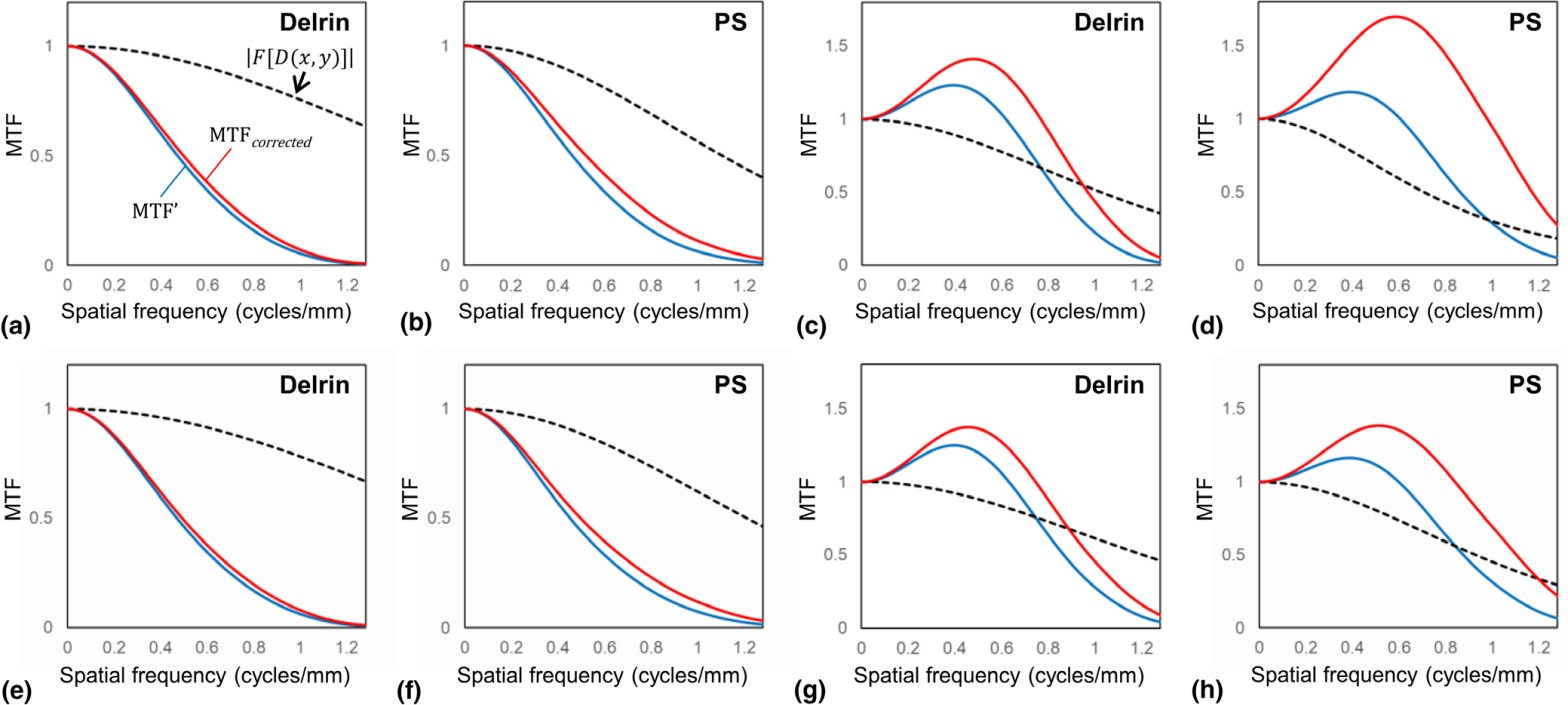
The modulation transfer functions and frequency characteristics of the deformation function $D\left(x,y\right)$ obtained for: IMR *Body Routine* level 1 using images of cylinders made of Delrin (a) and PS (b), *Body SharpPlus* level 1 using Delrin (c) and PS (d), *Body Routine* level 3 using Delrin (e) and PS (f), and *Body SharpPlus* level 3 using Delrin (g) and PS (h).

We measured ${\text{MTF}}^{{}^{\prime }}\left(w\right)$ using the circular‐edge technique with image averaging, and obtained ${\text{MTF}}_{\mathrm{corrected}}\left(w\right)$ by correcting ${\text{MTF}}^{{}^{\prime }}\left(w\right)$ using the frequency characteristics of $D\left(x,y\right)$ (Fig. 3). The difference between ${\text{MTF}}^{{}^{\prime }}\left(w\right)$ and ${\text{MTF}}_{\mathrm{corrected}}\left(w\right)$ obtained for the IMR *Body Routine* level 1 algorithm was subtle for cylinders made of Delrin and PS [Figs. 3(a) and 3(b)]. For the IMR *Body SharpPlus* level 1 algorithm, the difference between ${\text{MTF}}^{{}^{\prime }}\left(w\right)$ and ${\text{MTF}}_{\mathrm{correct}}\left(w\right)$ was clear [Figs. 3(c) and 3(d)]. These results for the corrections correspond to the frequency characteristic of each $D\left(x,y\right)$. The results for *Body Routine* level 3 and *Body SharpPlus* level 3 are shown in Figs. 3(e)–3(h), corresponding to those for level 1 in Figs. 3(a)–3(d). The different algorithm levels yielded similar results.

### 3.B Validation of MTF correction method

#### 3.B.1 **Validation using the averaged image** $\bar{I_{i}^{{}^{\prime }}}\left(x,y\right)$

**Figure 4:**
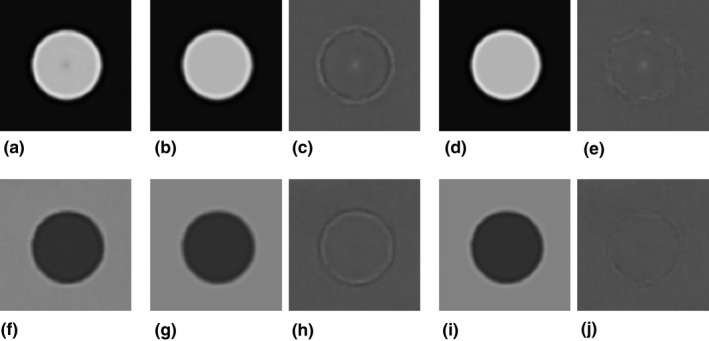
Results on IMR *Body SharpPlus* level 3 algorithm. (a) Mean image of 200 slice images of the cylinder (Delrin). (b) Image calculated using MTF' [Fig. 3(g)]. (c) Image obtained by subtracting the image in (a) from that in (b). (d) Image calculated using MTF*_corrected_* [Fig. 3(g)]. (e) Image obtained by subtracting the image in (a) from that in (d). (f–j) Results for PS cylinder. Images are analogous to those in (a–e), and the corresponding MTF' and MTF*_corrected_* are shown in Fig. 3(h). The window settings are WL/WW = 235/370 HU for images (a, b, d), 25/450 HU for images (f, g, i), and 0/200 HU for images (c, e, h, j).

**Figure 5:**
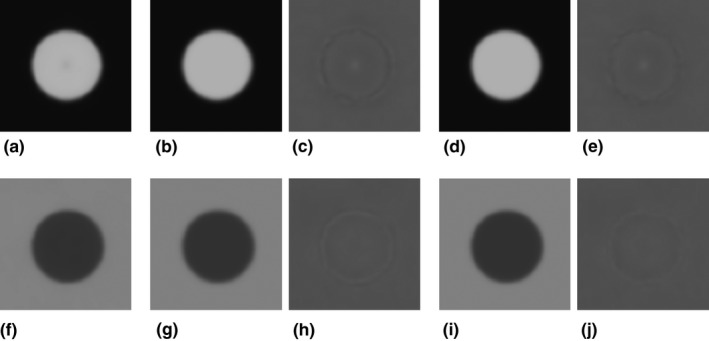
Results on IMR *Body Routine* level 3 algorithm. (a–j) Images are analogous to those in Fig. 4. The MTF' and MTF*_corrected_* used for calculating image (b) and (d) are shown in Fig. 3(e). The MTF' and MTF*_corrected_* used for calculating image (g) and (i) are shown in Fig. 3(f).

For the IMR *Body SharpPlus* level 3 algorithm, we compared the actual CT image $\bar{I_{i}^{{}^{\prime }}}\left(x,y\right)$ obtained by averaging Delrin cylinder images [Fig 4(a)] with the image calculated from the averaged effective object function $\bar{O_{i}^{{}^{\prime }}}\left(x,y\right)$ based on ${\text{MTF}}^{{}^{\prime }}\left(w\right)$ [Fig 4(b)], and subtracted $\bar{I_{i}^{{}^{\prime }}}\left(x,y\right)$ from the calculated image [Fig 4(c)]. In the same way, we calculated the image based on ${\text{MTF}}_{\mathrm{corrected}}\left(w\right)$ [Fig 4(d)], and subtracted $\bar{I_{i}^{{}^{\prime }}}\left(x,y\right)$ from the calculated image [Fig 4(e)]. The results for the PS cylinder images are shown in Figs. 4(f)–4(j), corresponding to those for the Delrin cylinder in Figs. 4(a)–4(e). The calculated images based on ${\text{MTF}}_{\mathrm{corrected}}\left(w\right)$ better matched the actual images for the edge regions compared with the calculated images based on ${\text{MTF}}^{{}^{\prime }}\left(w\right)$, as indicated by the subtraction images for both the Delrin and PS cylinders. The equivalent results for *Body Routine* level 3 are shown in Fig. 5. The RMSEs of these comparisons are shown in Table 1. The RMSEs resulting from using ${\text{MTF}}_{\mathrm{corrected}}\left(w\right)$ were smaller than those corresponding to ${\text{MTF}}^{{}^{\prime }}\left(w\right)$ under all conditions. For *Body Routine*, the difference between the actual image and the calculated images based on ${\text{MTF}}^{{}^{\prime }}\left(w\right)$ was minimal and therefore the effect of the MTF correction was lower than for *Body SharpPlus*.

**Table 1:** Root mean‐squared errors between the actual computed tomography image and calculated image based on modulation transfer function (MTF)

	Routine level 1		SharpPlus level 1		Routine level 3		SharpPlus level 3	
	Delrin	PS	Delrin	PS	Delrin	PS	Delrin	PS
MTF'	2.9	2.3	11.0	11.0	2.7	1.9	8.7	7.0
MTF*_corrected_*	2.5	1.7	4.8	3.8	2.4	1.6	4.0	2.8

#### 3.B.2 **Validation using each image** $I^{\prime }\left(x,y\right)$

**Figure 6:**
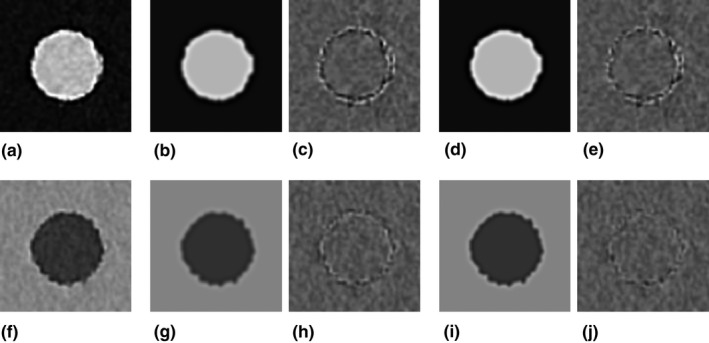
Results on IMR algorithm *Body SharpPlus* level 3. (a) One image of 200 slice images of the cylinder (Delrin). (b) Image calculated using MTF’ [Fig. 3(g)]. (c) Image obtained by subtracting the image in (a) from that in (b). (d) Image calculated using MTF*_corrected_* [Fig. 3(g)]. (e) Image obtained by subtracting the image in (a) from that in (d). (f – j) Results for PS cylinder. Images are analogous to those in (a – e), and the corresponding MTF’ and MTF*_corrected_* are shown in Fig. 3(h). The window settings are WL/WW = 235/370 HU for images (a, b, d), 25/450 HU for images (f, g, i), and 0/200 HU for images (c, e, h, j).

For the IMR *Body SharpPlus* level 3 algorithm, we compared an actual CT image of the Delrin cylinder [$I_{i=k}^{{}^{\prime }}\left(x,y\right)$ for *k* = 82, for example] [Fig. 6(a)] with the image calculated from the object function [$O_{i=k}^{{}^{\prime }}\left(x,y\right)$ for *k* = 82] based on ${\text{MTF}}^{{}^{\prime }}\left(w\right)$ [Fig. 6(b)], and subtracted $I_{i=k}^{{}^{\prime }}\left(x,y\right)$ from the calculated image [Fig. 6(c)]. In the same way, we calculated the image based on ${\text{MTF}}_{\mathrm{corrected}}\left(w\right)$ [Fig. 6(d)], and subtracted $I_{i=k}^{{}^{\prime }}\left(x,y\right)$ from the calculated image [Fig. 6(e)]. The results for the PS cylinder are shown in Figs. 6(f)–6(j), corresponding to those for the Delrin cylinder in Figs. 6(a)–6(e). The calculated images based on ${\text{MTF}}_{\mathrm{corrected}}\left(w\right)$ showed fewer differences in the edge regions from the actual images than the calculated images based on ${\text{MTF}}^{{}^{\prime }}\left(w\right)$, as indicated by the subtraction images for both the Delrin and PS cylinders. We calculated the RMSEs for these comparisons for all 200 slice images, and the average RMSEs are shown in Table 2. The average RMSEs corresponding to ${\text{MTF}}_{\mathrm{corrected}}\left(w\right)$ were smaller than those corresponding to ${\text{MTF}}^{{}^{\prime }}\left(w\right)$ under all conditions.

**Table 2:** Averaged root mean‐squared errors over 200 slice images between the actual computed tomography images and modulation transfer function (MTF)‐calculated images

	Routine level 1		SharpPlus level 1		Routine level 3		SharpPlus level 3	
	Delrin	PS	Delrin	PS	Delrin	PS	Delrin	PS
MTF'	16.0	12.4	49.4	41.9	13.5	9.4	35.3	23.9
MTF*_corrected_*	15.7	12.0	44.6	37.3	13.3	9.2	33.4	21.7

## 4 DISCUSSION

We considered that images reconstructed using the IMR algorithm potentially deform from the ideal object shape, depending on the noise in the edge regions (Fig. 1). This deformation is caused by the intrinsic nonlinearity of the IMR algorithms, and was not observed in FBP images. The edge shape deformation leads to errors in the MTF measurement when using image averaging with the circular‐edge technique. The calculated image based on the MTF (MTF’) measured by the circular‐edge technique showed differences from the actual (true) image in edge regions, especially when using the edge‐enhancement type IMR algorithm (*Body SharpPlus*) [Figs. 4(c), 4(h) and 6(c), 6(h)]; these results indicate the presence of errors in the MTF. Thus, the aforementioned issue is a pitfall of the circular‐edge technique. To address this issue, we made several assumptions, modified the equation for the image generating system, and proposed a method to correct the MTF. The calculated image based on the MTF corrected by our method (MTF*_corrected_*) showed fewer differences from the actual image under all conditions (Tables 1 and 2). We believe that these results demonstrate the validity of our assumptions and proposed method, although strict validation of our approach with respect to the IMR algorithm is precluded by the use of complicated (unknown) vendor‐specific approaches. When using edge‐enhancement type IMR algorithms, our proposed MTF correction method improves the results of the circular‐edge technique accompanied by image averaging.

Large deformations were observed in the images reconstructed using the algorithm *Body SharpPlus* compared with those reconstructed using *Body Routine* (Fig. 1). The deformations caused blurring in an average image, reducing the frequency characteristics of $D\left(x,y\right)$. As shown in Fig. 3, the values of the frequency characteristics of $D\left(x,y\right)$ for *Body SharpPlus* were smaller than those for *Body Routine*. By dividing by the frequency characteristics of $D\left(x,y\right)$, MTF values of *Body SharpPlus* were enhanced, and there was a noticeable shift in the peaks of frequency that led to a considerable difference between ${\text{MTF}}^{{}^{\prime }}\left(w\right)$ and ${\text{MTF}}_{\mathrm{corrected}}\left(w\right)$. In measurements of MTFs for edge‐enhancement and/or high‐resolution IMR algorithms, the effect of MTF correction using our method was high.

The proposed MTF correction method is performed using only the images already obtained for the circular‐edge technique, and requires neither further CT scanning nor an additional phantom preparation. We obtain the effective object functions $O_{i=k}^{{}^{\prime }}\left(x,y\right)$ using simple thresholding and morphological analysis, and obtain the mean effective object function $\bar{O_{i}^{{}^{\prime }}}\left(x,y\right)$ by averaging. The frequency characteristics of the deformation function $D\left(x,y\right)$ are determined from $\bar{O_{i}^{{}^{\prime }}}\left(x,y\right)$ based on Eq. (4) by applying the circular‐edge technique (described in Section 2. C. 2.). That is, the circular‐edge technique used for the MTF measurement is used again on the image $\bar{O_{i}^{{}^{\prime }}}\left(x,y\right)$, and no additional methods (programming) are required. Finally, the MTF is corrected by dividing by the frequency characteristics of $D\left(x,y\right)$, as indicated in Eq. (9). Thus, the implementation of the correction method is simple.

Our study has some limitations. First, we used a limited number of scan/reconstruction conditions and we used the cylinder phantom with two contrasts and one size. To increase the robustness of our conclusions, we plan to perform further investigations under various conditions (e.g., dose levels, IR algorithms, and various object contrasts/sizes in the phantom). Second, we applied a simple thresholding technique to generate the effective object functions. In preliminary experiments, we used edge detection techniques^20^, ^21^ to generate the effective object function, but they did not work well because of a low contrast‐to‐noise ratio, especially in PS cylinder images reconstructed using the *Body SharpPlus* level 1 algorithm. Our method, using a simple thresholding, provided a suitable effective object function (as indicated in Fig. 2), even when using a lower‐contrast object (the contrast of the PS to the background was approximately −130 HU). However, when using a considerably lower‐contrast object, improvements of generating the effective object function might be necessary.

## 5 CONCLUSION

We demonstrate a pitfall in the circular‐edge technique accompanied with image averaging for MTF measurement, particularly when using an edge‐enhancement type IMR algorithm. To address this issue, we made several assumptions, modified the equation for the image generating system, and proposed a method to correct the MTF. We confirmed the validity of the proposed method by comparing the calculated images based on the corrected MTF with the actual (true) images. When using an edge‐enhancement type IMR algorithm, the MTF correction method improves the results obtained using the circular‐edge technique.

## CONFLICT OF INTEREST

The authors have no relevant conflict of interest to disclose.

## References

[1] Price RG , Vance S , Cattaneo R 2nd , et al. Characterization of a commercial hybrid iterative and model‐based reconstruction algorithm in radiation oncology. Med Phys. 2014;41:081907. 25086538 10.1118/1.4885976

[2] Miéville FA , Gudinchet F , Brunelle F , Bochud FO , Verdun FR . Iterative reconstruction methods in two different MDCT scanners: physical metrics and 4‐alternative forced‐choice detectability experiments—a phantom approach. Phys Med. 2013;29:99–110. 22217444 10.1016/j.ejmp.2011.12.004

[3] Samei E , Richard S , Lurwitz L . Model‐based CT performance assessment and optimization for iodinated and noniodinated imaging tasks as a function of kVp and body size. Med Phys. 2014;41:081910. 25086541 10.1118/1.4890082 PMC4111837

[4] Löve A , Olsson ML , Siemund R , et al. Six iterative reconstruction algorithms in brain CT: a phantom study on image quality at different radiation dose levels. Br J Radiol. 2013;86:20130388. 24049128 10.1259/bjr.20130388 PMC3830436

[5] Geyer LL , Schoepf UJ , Meinel FG , et al. State of the art: iterative CT reconstruction techniques. Radiology. 2015;276:339–357. 26203706 10.1148/radiol.2015132766

[6] Li K , Tang J , Chen GH . Statistical model based iterative reconstruction (MBIR) in clinical CT systems: experimental assessment of noise performance. Med Phys. 2014;41:041906. 24694137 10.1118/1.4867863 PMC3978426

[7] Richard S , Husarik DB , Yadava G , Murphy SN , Samei E . Towards task‐based assessment of CT performance: system and object MTF across different reconstruction algorithms. Med Phys. 2012;39:4115–4122. 22830744 10.1118/1.4725171

[8] Chen B , Christianson O , Wilson JM , Samei E . Assessment of volumetric noise and resolution performance for linear and nonlinear CT reconstruction methods. Med Phys. 2014;41:071909. 24989387 10.1118/1.4881519

[9] Li K , Garrett J , Ge Y , Chen GH . Statistical model based iterative reconstruction (MBIR) in clinical CT systems. Part II. Experimental assessment of spatial resolution performance. Med Phys. 2014;41:071911. 24989389 10.1118/1.4884038 PMC4106476

[10] Takata T , Ichikawa K , Mitsui W , et al. Object shape dependency of in‐plane resolution for iterative reconstruction of computed tomography. Phys Med. 2017;33:146–151. 28089191 10.1016/j.ejmp.2017.01.001

[11] Yu L , Vrieze TJ , Leng S , Fletcher JG , McCollough CH . Technical note: measuring contrast‐ and noise‐dependent spatial resolution of an iterative reconstruction method in CT using ensemble averaging. Med Phys. 2015;42:2261–2267. 25979020 10.1118/1.4916802 PMC4401802

[12] Urikura A , Ichikawa K , Hara T , Nishimaru E , Nakaya Y . Spatial resolution measurement for iterative reconstruction by use of image‐averaging techniques in computed tomography. Radiol Phys Technol. 2014;7:358–366. 24880960 10.1007/s12194-014-0273-2

[13] Leipsic J , Labounty TM , Heilbron B , et al. Adaptive statistical iterative reconstruction: assessment of image noise and image quality in coronary CT angiography. AJR Am J Roentgenol. 2010;195:649–654. 20729442 10.2214/AJR.10.4285

[14] Singh S , Kalra MK , Hsieh J , et al. Abdominal CT: comparison of adaptive statistical iterative and filtered back projection reconstruction techniques. Radiology. 2010;257:373–383. 20829535 10.1148/radiol.10092212

[15] Ohkubo M , Wada S , Kunii M , Matsumoto T , Nishizawa K . Imaging of small spherical structures in CT: simulation study using measured point spread function. Med Biol Eng Comput. 2008;46:273–282. 17994259 10.1007/s11517-007-0283-x

[16] Ohkubo M , Wada S , Ida S , et al. Determination of point spread function in computed tomography accompanied with verification. Med Phys. 2009;36:2089–2097. 19610298 10.1118/1.3123762

[17] Polacin A , Kalender WA , Brink J , Vannier MA . Measurement of slice sensitivity profiles in spiral CT. Med Phys. 1994;21:133–140. 8164579 10.1118/1.597251

[18] Shih AY , Driscoll JD , Drew PJ , Nishimura N , Schaffer CB , Kleinfeld D . Two‐photon microscopy as a tool to study blood flow and neurovascular coupling in the rodent brain. J Cereb Blood Flow Metab. 2012;32:1277–1309. 22293983 10.1038/jcbfm.2011.196 PMC3390800

[19] Kayugawa A , Ohkubo M , Wada S . Accurate determination of CT point‐spread‐function with high precision. J Appl Clin Med Phys. 2013;14:3905. 23835372 10.1120/jacmp.v14i4.3905 PMC5714539

[20] Rathnayaka K , Sahama T , Schuetz MA , Schmutz B . Effects of CT image segmentation methods on the accuracy of long bone 3D reconstructions. Med Eng Phys. 2011;33:226–233. 21030288 10.1016/j.medengphy.2010.10.002

[21] Di Y , Li MY , Qiao T , Lu N . Edge detection and mathematic fitting for corneal surface with Matlab software. Int J Ophthalmol. 2017;10:336–342. 28393021 10.18240/ijo.2017.03.02 PMC5360765

